# Book Notices, Reviews, &c.

**Published:** 1856-03

**Authors:** 


### BOOK NOTICES, REVIEWS, &c.

SOME years ago, we had under our medical care a lady of great sweetness of character, the mother of a large family of children, to one of whom we sent a beautiful little book, beautiful in its mechanical execution, and as beautiful in sentiment, as it was true. Some days afterwards we inquired of the mother how her child liked the little book. "Really Doctor, I have not yet placed it in her hands, as I have not had an opportunity of reading it; for it is my habit, not to allow my younger children to read any book unless I have first examined it myself." This accomplished woman, with almost regal wealth at her command, had dismissed the world, with all the fascinations which a southern city can throw around social intercourse, and consecrated herself to the moral and religious tuition of her children. She soon passed away, but we feel quite sure, that she and the little ones she left behind her, will form a blest re-union above, a whole family saved in heaven, to be separated no more.

This lesson of example has its own loud and impressive teaching, and we pass on to speak as a physician of the ruinous physical, mental and moral tendencies which very many books have on boys and girls, on young women and young men. Many publications, even of what are called "*respectable*" publishing houses, are not more fit for young persons to read than *Don Juan* or the works of *George Sands*.

When children have become able to read with ease, their literary maws expand to most wonderful dimensions; they devour every book within their reach, which is adapted to their comprehension, and so eager are they to drink in knowledge sometimes, that play is forgotten, they have to be reminded at meal time that the table is waiting, and before you are aware of it, they have slipped away, and you find them in some retired corner, as deeply absorbed in reading, as was Newton in following out some of his great calculations. Physically, this habit is ruinous; the brain is exercised too much, stimulated too highly; it consumes the nervous energy which ought to have gone to the stomach, to be expended in the more perfect digestion and elimination of the essence of the food for the support of the body; presently the brain becomes overworked, loses its vital force, its capability of resisting disease; being overwrought, its vitality is below par, and the very first ailment the child has, cold, fever, any thing, "*goes to the brain*" in common phrase, and death is very generally inevitable. Thus perished within six months, the beautiful boy who took the premium at Barnum's Baby show: at that time, he was reported never to have been sick a day, and he was as bright as he was beautiful.

If the mother would make it a point to read carefully every book, previous to its being placed in the hands of her child, the result would most generally be, that very few books indeed, in the course of a year would the children have to read, because, household duties properly attended to, take up all a mother's time; the child then, not having many books, would give more time to play, and be that much more likely to live.

Parents are less careful than they would otherwise be, in placing books in the hands of their children, in consequence of the confidence they have in the newspapers which they take; they reason thus, "the editor speaks very highly of it; he received an early copy for examination, and says *it is well worth a place in every family.*" This parent does not know, that nine times out of ten, the editor never saw the book, or if he did, never read a dozen, not three consecutive pages; that as far as large towns and cities are concerned, the editor pays a man for reviewing books, and the owners of these books pay this same, or some other man to write these favorable notices; this same notice manufacturer being more frequently than otherwise some learned foreigner or some native literary adventurer, who, having some capabilities, and flattered into the belief that he has more, takes the very natural next step of cutting loose from the strict trainings of parental piety, and launches himself out into the world as a man of "*liberal views!*" is lauded by a certain class as being "entirely free from *cant* and old-fashioned puritanical notions;" in due process of time, this same young man is voted a *good fellow*, grows witty over a glass of "half-and-half," and anon we read ———— morning paper, "*Died yesterday, suddenly, of disease of the heart*" or "apoplexy," which being interpreted correctly, means "Brandy."

It is well known that such is the no uncommon history of itemizers and sub-editors, and upon their opinion is it, that we introduce books into our families, to supply the moral pabulum of our children.

We do not mean to say that a majority of the men who write the book notices for the secular press, are persons of this character, but *some* of them are, and this being the case, we contend that it is not safe for a parent to put a book into the hands of a child, on the recommendation of the secular press of cities and large towns, unless a previous examination authorizes the parent to endorse the sentiments of the reviewer. That the book notices of the secular press of cities and large towns are an unsafe guide in our estimate of new publications, we have only to recur to the unstinted praise from hundreds of presses, bestowed with such lavish profusion on the lustful, lecherous and adulterous issues of "*Hot Corn*," "*Mary Lyndon*," and the like.

There is another class of books, against which we warn all who are so happy as to have children, whom they daily hope and pray, will be their solace, when old age comes upon them; the titles of some of these corrupting publications read in this wise: "Anthropology," "Every Mother's Own Book," "Physiology," "*Woman's Manual*" ☞ *for every female's own private use!*" with colored plates. "Young Man's Adviser," &c. &c.—all such publications, with *Family Companion*, *Domestic Medicine*, and the like, had far better be thrown into the fire.

By reading such publications, young men have applied to us for ailments which these readings have caused them to magnify to the size of alarming maladies, and having spent hundreds of dollars on the authors of these books without being made a whit better, they have applied to us in a state of mind bordering on despair, which true views have permanently dissipated.

We are inclined to think, that all medical publications should be rigidly excluded from the libraries of the young, with a few exceptions, such as "Anatomy, Physiology and Hygiene, designed for academies, schools and families, by Calvin Cutter, M. D." Uses and Abuses of Air, by Dr. Griscom, of this city, and our own Journal. If the three works we have named were in every household in the United States, and were read and practised upon, even to a limited extent, we believe that a revolution would take place in public opinion, in reference to human health, which would add years to the life of the next generation.

In view of the very prevalent prostitution of the secular press to the interests of the large publishing houses of our country, we suggest that all "*book notices* and *reviews*" of the weekly press be considered a nuisance, which ought to be abated, and the sooner the better; and as a substitute, let a *succinct index of the subjects treated of*, be published without any opinion whatever. And as to the monthly and quarterly publications, we have long been of the opinion, that a simple statement of their contents, terms and place of publication, would be "valuable information" to readers, and would in very many instances, sell a copy or gain a new subscriber, where the most "splendid notice" would have failed to secure a second thought. We have in many instances purchased a book or paper or monthly on account of a single item of its contents. This suggestion merits more than a passing thought on the part of publishers and the press, and we believe the community would be a large gainer in a moral, as well as a literary point of view.

